# Towards Providing Solutions to the Air Quality Crisis in the Mexico City Metropolitan Area: Carbon Sequestration by Succulent Species in Green Roofs

**DOI:** 10.1371/currents.dis.bb66ae4f4f3c6eb118a019a29a9ce80f

**Published:** 2017-03-31

**Authors:** Margarita Collazo-Ortega, Ulises Rosas, Jerónimo Reyes-Santiago

**Affiliations:** Departamento de Biología Comparada, Facultad de Ciencias, Universidad Nacional Autónoma de México, Ciudad de México, México; Jardín Botánico, Instituto de Biología, Universidad Nacional Autónoma de México, Ciudad de México, México; Jardín Botánico, Instituto de Biología, Universidad Nacional Autónoma de México, Ciudad de México, México

## Abstract

**INTRODUCTION:**

In the first months of 2016, the Mexico City Metropolitan Area experienced the worst air pollution crisis in the last decade, prompting drastic short-term solutions by the Mexico City Government and neighboring States. In order to help further the search for long-term sustainable solutions, we felt obliged to immediately release the results of our research regarding the monitoring of carbon sequestration by green roofs. Large-scale naturation, such as the implementation of green roofs, provides a way to partially mitigate the increased carbon dioxide output in urban areas.

**METHODS:**

Here, we quantified the carbon sequestration capabilities of two ornamental succulent plant species, Sedum dendroideum and Sedum rubrotinctum, which require low maintenance, and little or no irrigation. To obtain a detailed picture of these plants’ carbon sequestration capabilities, we measured carbon uptake on the Sedum plants by quantifying carbon dioxide exchange and fixation as organic acids, during the day and across the year, on a green roof located in Southern Mexico City.

**RESULTS:**

The species displayed their typical CAM photosynthetic metabolism. Moreover, our quantification allowed us to conservatively estimate that a newly planted green roof of Sedum sequesters approximately 180,000,000 ppm of carbon dioxide per year in a green roof of 100 square meters in the short term.

**DISCUSSION:**

The patterns of CAM and carbon dioxide sequestration were highly robust to the fluctuations of temperature and precipitation between seasons, and therefore we speculate that carbon sequestration would be comparable in any given year of a newly planted green roof. Older green roof would require regular trimming to mantain their carbon sink properties, but their carbon sequestration capabilities remain to be quantified. Nevertheless, we propose that Sedum green roofs can be part of the long-term solutions to mitigate the air pollution crisis in the Mexico City Metropolitan area, and other “megacities” with marked seasonal drought.

## Resumen

INTRODUCCIÓN: En los primeros meses del 2016, la Ciudad de México y su zona metropolitana conurbada ha experimentado una de las peores crisis de contaminación del aire de la última década, urgiendo a las autoridades a tomar medidas drásticas a corto plazo. A fin de proporcionar elementos para investigaciones de sostenibilidad a largo plazo quisimos dar a conocer nuestros resultados de monitoreo de captura de carbono por plantas de azoteas verdes. Estas azoteas son una forma de naturación a gran escala, que constituyen una alternativa para mitigar parcialmente el incremento de dióxido de carbono en las áreas urbanas.

MÉTODOS: Nosotros cuantificamos la capacidad de captura de carbono por dos especies de suculentas ornamentales, *Sedum dendroideum* y *Sedum rubrotinctum*, que requieren bajo mantenimiento y nula o poca irrigación. Para tener una idea detallada de la capacidad de captura, medimos el carbono tomado por las plantas durante el día por un año en plantas de *Sedum* localizadas en una azotea verde en el sur de la Ciudad de México.

RESULTADOS: Estas especies presentan el metabolismo fotosintético CAM. La cuantificación obtenida nos permite estimar que una azotea verde de *Sedum* de 100 m^2^ captura aproximadamente 1.8 x 10^8^ ppm de CO_2_ por año.

DISCUSIÓN: Los patrones de CAM y de captura de CO_2_ fueron bastante robustos a las fluctuaciones de temperature y precipitación estacionales, por lo cual especulamos que la captura de carbono sería similar en cualquier otro año de una azotea verde de reciente plantación. Azoteas de mayor edad requerirían podas regulares para mantener sus propiedades de captura de carbono, sin embargo, no se sabe qué capacidad pueden tener para capturar carbono. No obstante, proponemos que las azoteas verdes plantadas con *Sedum* son una parte de la solución a largo plazo para mitigar las crisis de contaminación del aire en la zona metropolitana de la Ciudad de México, y de las megaurbes con una marcada estación de sequía.

## Author Contributions

MCO & JRS designed the project; MCO performed experiments; MCO and UR analyzed results; MCO & JRS provided materials and reagents to perform experiments; MCO & UR wrote the manuscript. MCO: orcid.org/0000-0001-6618-4920; UR: orcid.org/0000-0001-5088-2679

## Corresponding Authors

MCO (mague.collazo@ciencias.unam.mx) and UR (urosas@ib.unam.mx)

## Keywords

Plant physiology, Crassulacean Acid Metabolism, urban biology, megapolis, environmental crisis, plant urban physiology, *Sedum dendroideum*, *Sedum rubrotinctum*, air pollution, bioremediation

## Competing Interests statement

The authors have declared that no competing interests exist.

## Data Availability statement

Raw data available on doi:10.6084/m9.figshare.3395887

## Introduction

Deforestation, urbanization and the intensive use of fossil fuels are some of the main causes of the increase of carbon dioxide (CO_2_) since the Industrial Revolution in the nineteenth century, contributing to the current changes in climate 1. These CO_2_ levels have gone from 280 ppm to 380 ppm, and it is estimated that levels could reach 770 ppm by the end of the twenty-first century 2. This dramatic increase in CO_2_ levels, particularly in urban regions, has caused a change in the global carbon cycles 2^,^^3^. Thus, vegetation plays a crucial role in urban ecosystems, by sequestering carbon excess, storing carbon as biomass, and releasing oxygen and water vapor through evapotranspiration. In recent decades urban ecosystems have drastically expanded and increased in complexity due to mass changes in economic activities, the migration of people from rural environments to cities 4, and widespread use of motorized vehicles. Unplanned growth of urban areas sometimes produces “megacities” where space becomes a luxury, and vegetation is often sacrificed to give way to urban infrastructure.

This is the case of Mexico City and its Metropolitan Area, otherwise known as Zona Metropolitana del Valle de México (ZMVM). This is the fourth most populated metropolis of the world and the largest in Latin America 5^,^^6^, with more than 20 million inhabitants according to a 2010 census (INEGI, http://www.inegi.org.mx). According to the Mexican Fifth National Communication to the United Nations on Climate Change, CO_2_ emissions increased 23.6 % from 1990 to 2010, coming mainly from fossil fuels, land use changes, and lack of silviculture. In the first third of 2016, the ZMVM is experiencing the worst environmental crisis of air quality of the last decade, demonstrating that short-term solutions implemented since the 1980s when the air quality became extremely critical, have been insufficient. In 2009, the Dirección de Reforestación Urbana, Parques y Ciclovías de la Secretaría de Medio Ambiente del Distrito Federal (today Mexico City), pointed out that poor metropolitan growth planning, deficient management of green areas, lack of legislation on urban vegetation, and poor conservation efforts, have all contributed to the deterioration of the green urban landscape, and their associated environmental and social benefits. In addition to better management of public green spaces, it is necessary to urgently reassess the strategies towards sequestering carbon and other air pollutants. To archive this, the mega-polis inhabitants must play an active role in tackling the challenge.

Besides reducing gas emissions from fossil-fuel combustion, in other cities the promotion of urban vegetation has been adopted as a method to eliminate pollutants and purify the air 7^,^^8^. Because of the scarcity of space in large cities, a strategy to increase the vegetation cover is through the implementation of what are known as green roofs, eco-roofs, living roofs, or roof gardens . Green roofs are defined as any rooftop covered by layers of roof coating, plant substrate, and vegetation 9. Green roofs have been associated with multiple environmental, ecological, health, and aesthetic benefits 3^,^^9^^,^^10^^,^^11^^,^^12^^,^^13^^,^^14^^,^^15^. Despite the relevance of green roofs as a potential solution to improve air quality in “megacities,” there is scarce experimental data in planta on the capabilities of green roofs to uptake CO_2_. Therefore, quantifying the green roofs’ capacity to sequester and retain CO_2_, pollutants, and other greenhouse gases is imperative under the current environmental crisis scenario, particularly in the ZMVM.

Currently there is robust evidence supporting the use of plants to capture air pollutants. However, we still need in planta qualifications on the role of green roofs to capture pollutants 16. NO_x_, SO_2_, PM_10_ and O_3_ are among the pollutants that have been quantified as sequestered by green roof vegetation 7^,^^17^^,^^18^^,^^19^. Experimentally, CO_2_ green roof-uptake quantification seems to be rather challenging, and mostly estimated using proxies, such as biomass accumulation 13^,^^20^, models of carbon-oxygen balance 21^,^^22^, macro-estimations of vegetation cover in large cities 7^,^^13^^,^^23^ or methods more appropriate for urban forests, rather than herbaceous plants 17.

In their studies measuring carbon uptake on green roofs, Li *et al* (2010) measured the CO_2_ uptake in a green roof, placing the IRGA within grass-shrubs and comparing it to a location outside the shrub, obtaining an average of 12.9 mg C m^-3^ per day 8. Ondoño et al (2016) estimated carbon and nitrogen uptake by quantifying the element composition on a green roof of weedy and grassy plants, obtaining 36.52 g C m^-2^ at the end of 9 months 24. Regarding *Sedum* green roofs, Getter *et al* (2009) quantified carbon storage in green roofs of several *Sedum* species, by measuring accumulated dry matter in the shoot, root, and soil, obtaining 160 g C m^-2^ during a two year period 13. More recently, Whittinghill *et al* (2014) measured carbon content and dry weight, and found that *Sedum* green roofs captured 1940 g C m^-2^ in a 14-month period and 3910 g C m^-2^ in a 12-month period 20. Overall, previous studies support the use of green roofs, and particularly, of highly resilient *Sedum* species to improve air quality conditions, in particular in overcrowded urban areas. However, we still lack data on the carbon uptake by *Sedum* plants, measuring CO_2_ exchange.

During the first months of 2016, the ZMVM has experienced one of the worst air quality crises since the 1980s. This has forced the local governments of Mexico City and surrounding States to implement extreme measures, such as emergency “No Driving Today Program”. However, these are often short-term and unsustainable solutions, because they promote for instance, the acquisition of a second car by the inhabitants of the ZMVM. In light of the current air pollution environmental crisis, we felt compelled to release our monitoring of carbon uptake in a green roof in Southern Mexico City, populated with low-maintenance, low irrigation-requirement, crassulacean species of *Sedum*. Our results show the potential of the *Sedum* green roofs as a self-sustainable solution. We hope these results help policy makers of the ZMVM and other urban areas with similar environmental conditions to implement informed long-term solutions towards tackling air quality crises.

## Methods

**Green roof location. **The green roof was located on the Southwestern area of Mexico City. This green roof was installed in 2009, comprising a total area of 42 square meters, at the Jardín Botánico, Instituto de Biología, Universidad Nacional Autónoma de México, Delegación Coyoacán, within the Reserva Ecológica del Pedregal de San Ángel (REPSA; http://www.repsa.unam.mx) (19° 13' 45'' N and 99° 19' 37'' W), 2330 m.a.s.l. (Figure 1A-C), and subtropical highland climate. The highest temperatures are from March to May (16-19° C), and the lowest are from December to February (12-14° C), with a mean annual temperature of 15.6°C. Precipitation follows a seasonal pattern: the rainy season occurs from June to October, while the dry season is from November to May, with an annual mean precipitation of 833 mm 25.

**Figure:**
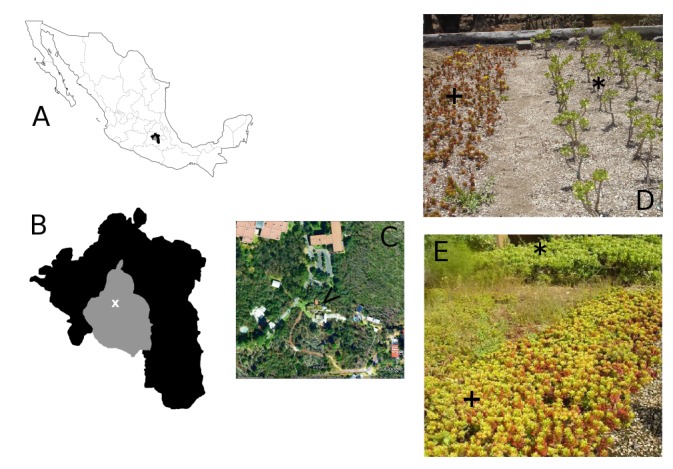
**Figure 1.** Green roof location within the Jardín Botánico of the Universidad Nacional Autónoma de México; **A.** Map of Mexico; **B.** Zona Metropolitana del Valle de México (ZMVM), according to Instituto Nacional de Estadística y Geografía (INEGI, México), x denotes the location of the Jardín Botánico; **C.** Google maps view of the Jardín Botánico, arrow (<) denotes the location of the green roof; **D. ***Sedum* green roof at the start of the experiment (1-month old plant-cuts); **E. ***Sedum* green roof towards the end of the experiment (11-month old plants). *Sedum dendroideum, +*Sedum rubrotinctum*.

**Green roof construction.** A completely flat roof was leveled up with concrete and bordered with 10 cm-tall edges (cost $7.5 USD per m^-2^). The roof was then insulated with a waterproof layer ($0.40 USD per m^-2^), an insulated asphalt layer ($15 USD per m^-2^), and a root-proof mesh ($16 USD per m^-2^). On top of these insulating and protecting materials, a 10 cm deep layer of substrate was uniformly placed, composed of tepojal (to facilitate drainage) and compost (for organic nutrients) in a ratio 1:1 ($11 USD per m^-2^). It is also estimated that initial plant cuttings cost $12.5 per m^-2^. All costs are expressed in US dollars as of November 2016. Green roofs require trimming of the excess of plants two years after the initial planting, and every 6 months thereafter. Every trimming removes approximately 30% of the old foliage.

Regarding the soil, we measured the soil apparent and actual densities, pH, electrical conductivity, percentage of moisture and texture 26^,^^27^. The bulk density was 1.045g cm^-3^, organic type, 13.12% humidity, pH 6.05, category moderately acidic, conductance 0.127 S m^-1^, 27.1 % clay, 12.4% lime, and 72.4% sand, which corresponds to a loam soil.

**Biological subjects**: We used the crassulacean species *Sedum dendroideum* Moc. Sessé ex DC. and *S. rubrotinctum* R.T. Clausen. *S. dendroideum* is a bushy plant of 50-100 cm in height, and up to 100 cm in diameter. In Mexico, its populations are distributed along the Trans-Mexican Volcanic Belt, and surrounding areas on the states of Hidalgo, Puebla, Estado de México, Oaxaca, and Querétaro 28. *S. rubrotinctum* has a herbaceous lifestyle, of 15-20 cm in height, heavily branched at the base, originally from the East, South, and Center of Mexico, but thought to be a cultivated hybrid of *S. stahlii* and *S. pachyphylum* 28. The cuts were obtained from 10 year-old parental plants at another green roof, at the Jardín Botánico de la Universidad Nacional Autónoma de México. *S. rubrotinctum*’s cuts were 10-15 cm tall, and *S. dendroideum*'s were 20-30 cm tall. Cuts were disinfected with Terramycin 1 g L^-1^ for 24h, and TRICON fungicide. Before planting, all cuts were treated at the base with Radix 10000 ppm to promote root formation. 72 cuts of each *Sedum* species were planted, every 30 cm in *S. dendroideum* and 10 cm in *S. rubrotinctum*, giving a density of 3.4 and 10 plants per square meter, respectively. Planting was done in November 2009, and measurements were recorded from November 2009 to November 2010. At end of the experiment the vegetative growth of both *Sedum* species gave a 100% cover of the studied green roof area.

**CO_2_ uptake.** Quantification of CO_2_ uptake was performed using an Infrared Gas Analyzer (IRGA) (Qubyt Systems, Canada), which is a device designed to study gas exchange on flat leaves. Thus to quantify the CO_2_ uptake in succulent plant specimens, we evaluated gas exchange using glass jars. We initially used a glass jar of 418 mL connected with plastic tubes to the IRGA, so we could introduce the entire plant into the jar and seal any openings. As plant size increased, we used a larger glass jar of 1200 mL during the months of April to November 2010. After the month of June, we were only able to measure the part of the plant that fitted into the 1200 mL jar. Measurements were taken monthly on three plants per species randomly chosen at 0:00, 0:40, 1:20; 6:00; 6:40; 7:20; 12:00; 12:40; 13:20; 18:00; 18:40 y 19:20h. The IRGA was calibrated 10 mins prior every measurement, and the quantification of CO_2_ uptake took 15 mins in each case. The baseline of CO_2_ in the air was subtracted to the readings, and the differential was considered as the net CO_2_ uptake by any given plant. In addition we quantified the concentration of organic acids, on the same plants quantified for CO_2_ uptake, by dissecting the tissues and keeping them chilled until they arrived to the lab. The quantification of organic acids was done according to the titration protocol 29^,^^30^, which was done as follow: 1) 2g of tissue were dissected from leaves in each sample, tissues were grinded with 20mL of 50% methanol; 2) the ground tissue was filtered, and 10mL more of distilled water were added; 3) titration was done using a potentiometer, by adding sodium hydroxide 0.01N under constant agitation until a pH of 9 was reached. The added base was used to calculate the acidity per gram of fresh tissue. Raw data available on doi:10.6084/m9.figshare.3395887

**Statistical analysis. **We used a two-way Analysis of Covariance Variance (ANCOVA) to identify significant differences in CO2 uptake (ppm) due to month and species, and time of the day (time) as a covariate, according to the following model: *y_ppm_* = *Ɓ_month_* + *Ɓ_species_* + *Ɓ_time_* + *ɛ*. The analysis was done in JMP v12 (JMP, SAS, USA).

## Results

Succulent plants, particularly *Sedum* species, have been extensively used in green roofs because of their resistance to prolonged droughts, high temperatures, and strong winds 10^,^^13^^,^^31^. Here, we studied the *Sedum* species *Sedum dendroideum* Mociño & Sessé ex De Candolle, and *S. rubrotinctum* R. T. Clausen (Family Crassulaceae). These species have xerophytic morphology and physiology that allows them to cope with extremely dry environments. To assess the carbon capture of *Sedum* species in a green roof located in Southern Mexico City, we evaluated a plantation of 42 square meters, measuring CO_2_ exchange using an IRGA system, during the day, over a period of 12 months in 2009-2010. The highest carbon capture in both species was recorded during the night, with a peak at 6:00 (Figure 2). Over the periods of time that were measured, the total CO_2_ uptake was 2472 ppm (±40.35) from 0:00 to 7:20h, and 152 ppm (± 6.82) from 18:40 to 19:20h in *S. dendroideum*. Over the same intervals, *S. rubrotinctum* CO_2_ uptake was 1823 ppm (±40.70) from 0:00 to 7:20h, and 71 (±4.58) from 18:40 to 19:20h. In both species from 12:00 to 18:00 no CO_2_ uptake was recorded in most months, but the period of highest CO_2_ uptake was at 6:00h, with 1602 ppm (±11.05) and 1315 ppm (±14.49) on *S. dendroideum* and *S. rubrotinctum* correspondingly. The patterns of CO_2_ uptake on these *Sedum* species resemble the Crassulacean Acid Metabolism (CAM) carbon fixation patterns.

**Figure:**
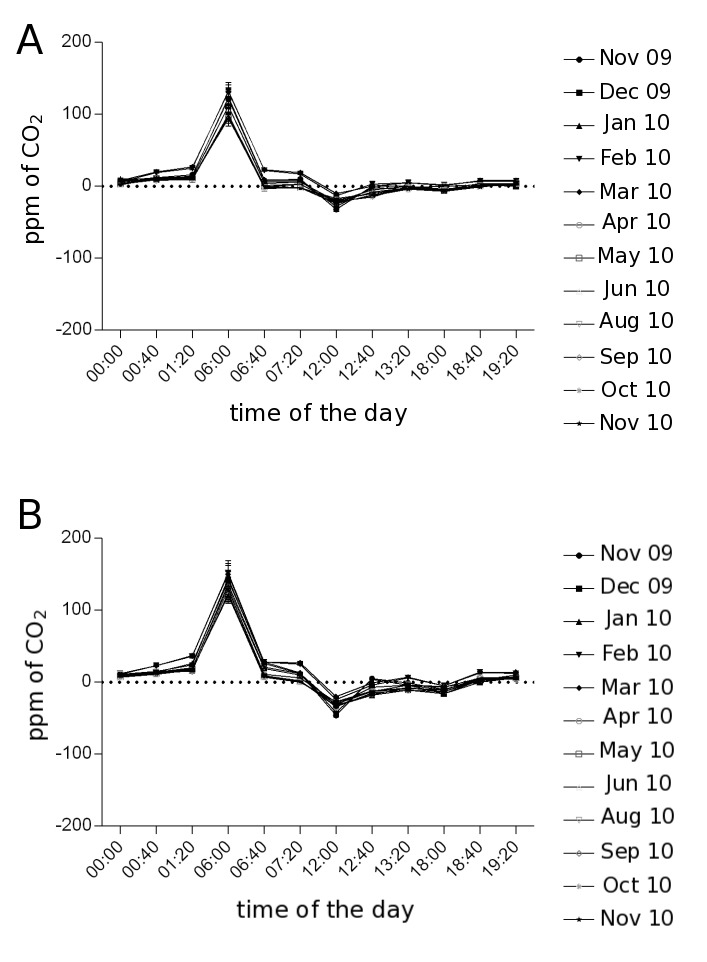
**Figure 2.** CO2 uptake in (A) *Sedum dendroideum* and (B) *Sedum rubrotinctum*, during the year, and across the day. Error bars: standard error, n = 3. Raw data available on doi:10.6084/m9.figshare.3395887

In order to validate our observations of CO_2_ uptake, during the experiment we simultaneously collected tissue from the measured plants to quantify the organic acid concentration using the titration method in the lab. The quantification of organic acids (Figure 3) confirmed our CO_2_ uptake observation regarding the CAM pattern, also indicating that S. dendroideum and S. rubrotinctum are strict CAM because they uptake most of the CO_2_, and accumulate organic acid during the night, but not during the day 32.

**Figure:**
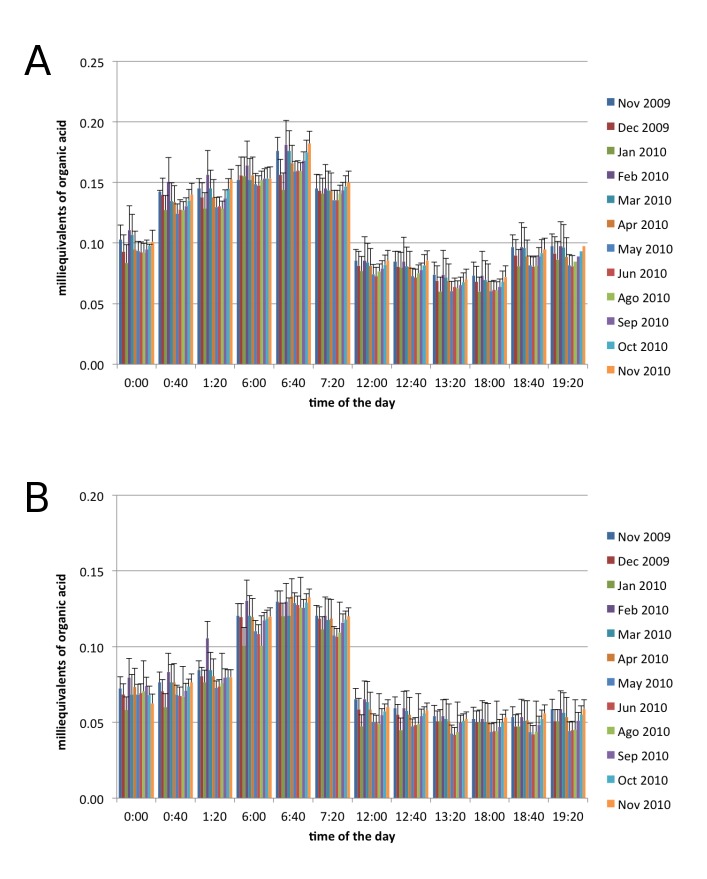
**Figure 3.** Quantification of organic acids in (**A**) *Sedum dendroideum* and (**B**) *Sedum rubrotinctum*, during the year, and across the day. Error bars: standard error, n = 3. Raw data available on doi:10.6084/m9.figshare.3395887

To further investigate the sources of variation on CO_2_ uptake during our experiment, we performed a 2-way ANCOVA, of carbon uptake (ppm) as a response of months, and species, and time of the day (time) as a covariate, according to the model *y_ppm_* = *Ɓ_month_* + *Ɓ_species_* + *Ɓ_time_* + *ɛ*. We found no statistical differences between months (F_(df 11)_ = 1.60, *p*=0.09), or species (F_(df 1)_= 1.74, *p*=0.18), but time of the day explained most of the variation in CO_2_ uptake (F_(df 1)_ = 104.37, *p*<0.0001). The subtle but not significant differences between *Sedum* species might be due to their distinctive life forms. *S. dendroideum* grows erect and bushy, whereas *S. rubrontinctum* grows herbaceous crawling the floor (Figure 1D-E).

Although it was not statistically significant, February and March were the months with the highest CO_2_ uptake, coincidentally some of the months with the most chance of high air pollution in the ZMVM. This observation illustrates the physiological plasticity of *Sedum* species, in which carbon sequestration might be influenced by environmental factors, such as temperature and precipitation (Table 1).

**Table 1:** 

Table 1. Measured net carbon capture of Sedum species in a green roof of Mexico City in a given day per month. Calculated from adding all the 15 mins measured intervals, and averaged from three plants, ± standard error. T_max_: maximum monthly temperature, T_min_: minimum monthly temperature; T_mean_: mean monthly temperature; ppt: monthly precipitation. Raw data available on doi:10.6084/m9.figshare.3395887						
Month	S. dendroideum	S. rubrotinctum	T_max_	T_min_	T_mean_	ppt
November 2009	193 ± 35.76	141 ± 13.89	21.9	8.9	15.4	3.4
December 2009	150 ± 19.67	118 ± 13.71	20.0	7.0	13.0	26.1
January 2010	100 ± 14.89	71 ± 16.29	21.4	7.2	14.0	69.0
February 2010	275 ± 23.05	233 ± 18.09	24.6	9.1	17.2	5.1
March 2010	284 ± 21.13	240 ± 15.12	26.4	10.8	18.5	21.9
April 2010	108 ± 15.33	75 ± 7.03	28.7	13.3	20.7	27.8
May 2010	114 ± 15.24	87 ± 8.69	27.1	14.5	19.7	95.0
June 2010	115 ± 14.58	84 ± 7.05	23.5	14.0	17.9	255.7
August 2010	144 ± 16.09	105 ± 1.06	23.8	14.0	18.8	133.9
September 2010	145 ± 15.90	102 ± 9.69	23.3	13.6	17.7	99.7
October 2010	150 ± 19.05	120 ± 12.21	23.7	9.6	16.2	3.5
November 2010	192 ± 18.39	138 ± 14.43	24.7	7.3	15.2	3.0

The carbon sequestered by *Sedum* plants can be partitioned into the shoot or root parts of the plant. In order to estimate the biomass partition in the studied species, in a parallel experiment with three plants each, we measured the dry biomass of plants 325 days after planting on a green roof. The largest fraction of the dry biomass was recorded in the shoot (Figure 4). The root:shoot ratio was 0.41 in S. dendroideum, and 0.15 in S. rubrotinctum, which are similar ratios reported for other succulent plants from arid environments 34. This shows that most of the biomass is allocated towards the succulent shoot. However, it is known that roots can produce CO_2_ because of root respiration and decomposition. The dynamics of carbon sequestration and decomposition in the root and the soil remains to be quantified, however, it is argued that in the short term this ecosystem could be an important sink of carbon 13. Therefore, to mantain a green roof as a carbon sink, we trim about 30% of the old foliage two years after the initial planting, and every six months thereafter. We especulate that trimming practice maintains the plant community as a carbon sink, and reduces carbon emission by decomposition. Nevertheless, carbon sequestration dynamics of older green roofs remain to be quantified.

**Figure:**
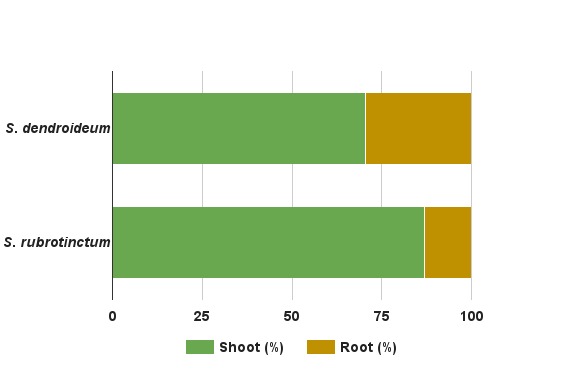
**Figure 4.** Dry biomass partition in *S. dendroideum* and *S. rubrotinctum* of plants (n=3) measured 325 days after planting on a green roof. The dry biomass of* S. dendroideum* was 70.3% in the shoot and 29.6% in the root (standard error ±3.1), whereas *S. rubrotinctum* was 86.8% in the shoot and 13.1% in the root (standard error ±1.1).

It is possible that carbon sequestered by *Sedum* species is allocated by the plant to the shoot or the root. The shoot adopts a perennial life-style, in which the leaves are succulent, permanently green, and only after several years, they senesce. This is why we believe that shoot senescence would have a small contribution to the re-emission of carbon to the environment. The biomass accumulated in the root, however, has the potential to contribute to the emission of carbon to the atmosphere by decomposition and degradation of the organic matter. Among desert perennial plants, succulents have the lowest root to shoot ratio, ranging from 0.04 to 0.15, which are dramatically smaller than desert shrubs that range from 3.2 to 7.3 33^,^^34^. In other words, the root biomass is disproportionately smaller. Moreover, an adaptation of succulent plant roots is to maintain roots, dehydrate them during the drought season and resurrect them during the new rainy season.

Our aim was to estimate the carbon sequestration capacity that green roofs can have. Therefore we used the data that we obtained from measuring individual plants during the day, and across the year to estimate the overall CO_2_ capture by *Sedum* plants. To do this we considered the average CO_2_ uptake from the three measured plants at each time, and month. Assuming that from 3 am to 6 am, *Sedum* plants have a steady carbon uptake similar to the 6am rate (see Figure 2), averaged the two species and multiplied our estimate of carbon uptake in 15 mins (period of IRGA quantification), by the total number of minutes in 3 hours, and obtained the carbon uptake in a given day per each month. This number was then multiplied by the number of days in any given month. Then we summed the months to estimate the total carbon uptake per plant during a year (July was assumed to have a mean value between June and August, and we averaged the two measurements of November months), giving 524,040 ppm of CO_2_ per plant per year, or 371.27 g C m^-2^. Furthermore, assuming that all the plants in the green roof have similar metabolic rates, we can estimate the amount of carbon that was sequestered in the studied green roof, by multiplying the carbon estimate per plant per year, by the total number of plants in our green roof. This gave a total of 75,461,760 ppm of CO_2_ in the 2009-2010 period of study in a green roof of 42 square meters, with 144 *Sedum* plants. In other words, a green roof of *Sedum* in Mexico City of 100 square meters, has the capacity to sequester 179,670,857 ppm of CO_2_. This estimate is highly conservative given that the number of plants per unit of area increases dramatically over the year as a result of vegetative growth (see Figure 1D-E). Remarkably, our estimation of a *Sedum* green roof of 100 square meters is equivalent to approximately 46% of the annual CO_2_ emission of a standard car (up to 7.5 km L^-1^) 35, driving every day 10km during 365 days, and estimated to produce 388,360,000 ppm CO_2_.

## Discussion

In this work we evaluated the capacity of two crassulacean *Sedum* species for capturing CO_2_, when grown in a green roof, during the day, and across the year. The months of February and March showed the maximum CO_2_ capture, 75% more than the summer month of June, a response that was possibly influenced by the temperature and precipitation patterns of the ZMVM. Consistent with the CAM, the highest CO_2_ uptake and accumulation of organic acids occurred early in the morning. We estimated that a green roof of *Sedum* of 100 square meters is able to sequester approximately 1.8 x 10^8^ ppm of CO_2_ per year (or 180,000 g C m^-2^) in the ZMVM. Our quantifications are within the range of previous studies in *Sedum* species, which range from 160 g m^-2^ over two years 13, and 1940-3910 g C m^-2^ over a 12-14 month period 20. It is important to remark that *Sedum* plants were not irrigated, making these species an ideal candidate, with low irrigation and maintenance requirements, towards promoting naturation efforts in “megacities”.

In our current scenario, carbon concentration in the air is increasing because of the intensive use of fossil fuels. This phenomenon is tightly linked to the growth of human populations and the loss of vegetation in cities, making the design of sustainable urban areas a challenging task. An example of a metropolitan area with poor air quality, linked to decades of deficient urban planning, and short-term solutions to address the air quality issue, is the ZMVM. Particularly, in the early months of 2016, the ZMVM is facing the worst air pollution crisis since the 1980s, which is evidence of the lack of long-term and sustainable environmental policies. Therefore, there is an urgent need to increase the capabilities to fix carbon and other pollutants, in order to improve the air quality in metropolitan areas such as the ZMVM. The widespread installations of green roofs, together with citizen involvement are part of the potential solutions to increase the CO_2_ sequestration in cities. The green roof system was first developed in Germany in the 1960s 3, and later adopted in several countries such as USA, Canada, and European countries 13^,^^36^. Despite their widespread implementation in developed countries and their relevance to tackle air pollution, the understanding of carbon and pollutant dynamics in green roofs is still scarce.

Green roofs are subject to extreme environmental conditions because of the lack of rainfall during certain periods of time, high temperatures, and intense wind. It is because of this that only few species have been used on green roofs, *Sedum* being the main species worldwide 10^,^^31^. As a CAM species, *Sedum* has fewer capabilities to capture CO_2_ than C_3_ or C_4_ plants; yet, *Sedum* species are often preferred because of the xerophytic lifeform, and their resilience to extreme conditions.

Carbon sequestration measured in our study is only representative of the environmental conditions of the period 2009-2010. Although we did not measure carbon sequestration in multiple years, we speculate that the pattern should be similar in any given year provided that the variation in temperature and precipitation during 2009-2010 studied period is larger than the corresponding parameters over several years 2005-2015 (CONAGUA, Mexico, 2016; http://www.gob.mx/conagua) (Figure 5). It is remarkable that, despite the extent of variation in temperature and precipitation, the patterns of CAM and carbon sequestration are highly robust during the period of study (Figure 2-3). Therefore, we believe that the estimated parameter of carbon sequestration can be extrapolated to multiple years with similar environmental conditions in the ZMVM. Moreover, our results further support the use of *Sedum* in green roofs of the ZMVM, as a highly resilient species to temperature and drought.

**Figure:**
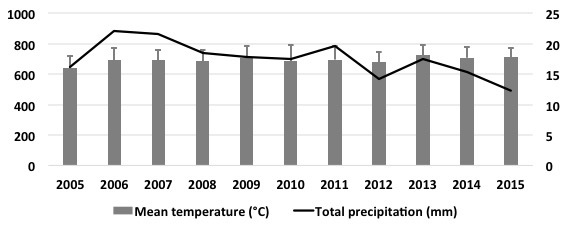
**Figure 5.** Historical climate in the Zona Metropolitana del Valle de México (ZMVM) from 2005 to 2015. Data obtained from Servicio Meteorológico Nacional, Comisión Nacional del Agua, México (CONAGUA, 2016).

In our study we estimated the carbon uptake of *Sedum* species at interval periods during the day and at chosen days of each month, giving a total CO_2_ uptake quantification time of 122.4 hours. We acknowledge that our study did not monitor the entire 24h, or every single day in any given month; however, we believe that our sampling strategy and the clear recapitulation of the CAM physiology during the day and across the year, makes our data robust enough to make projections of carbon uptake during the 24h, of the 365 days of the year. We consider that such quantification and projection of the CO_2_ uptake can be highly valuable information for policy makers, especially those who might be currently planning how to address the air pollution crisis of the ZMVM.

As expected, both *S. dendroideum* and *S. rubrotinctum* displayed a strict CAM photosynthesis, because they captured CO_2_ during the night, and early hours of the morning, including the dry (November to May) and wet (June to October) seasons. Remarkably, the plants only received an initial irrigation, and were later only watered by natural rainfall, which emphasizes their sustainability in “megacities” such as the ZMVM with seasonal droughts (Table 1). Moreover, *Sedum* is a perennial species, reducing the requirement for replanting year after year. Other perennials have been used on green roofs, for instance trees of up to 3m, which can have higher rates of carbon capture. However, trees often require costly maintenance and frequent irrigation. Trees can sequester more carbon at the global level, but as Getter et al (2009) pointed, “due to building weight restrictions and cost, shallow substrate extensive green roofs are more common than deeper intensive roofs” 13. Therefore we strongly support the use of *Sedum* species, together with other C_3_ or C_4_ plants, towards the widespread implementation of green roofs.

In summary, the high survival, fast growth, low or null-maintenance, low or null irrigation requirement, and capacity to capture CO_2_ make *Sedum* an ideal species for green roofs, as an alternative strategy for promoting vegetation in megacities with marked seasonal drought. Particularly, *Sedum* species would be an ideal solution for the ZMVM, which is in urgent need of long-term solutions towards tackling the air quality crisis of the last decades.
